# News and Miscellany

**Published:** 1901-11

**Authors:** 


### News and Miscellany.

**Dr. W. A. Harper,** of Austin, is at the New York Eye and Ear Infirmary, taking a special course in his specialty and attending the eye and ear clinics.

**The New Medical Practice Law of Texas** can be had in pamphlet form for 20 cents, stamps, by application to this office. TEXAS MEDICAL JOURNAL (the "Red-Back").

**Knocked Out.**—The Texas Legislature at its recent session knocked out of the appropriation bill the salary of the Secretary of the Quarantine Department, thus abolishing that office. Dr. I. J. Jones was the Secretary.

**Camp Treatment of Consumption.**—Dr. Cary H. Wilkinson of Galveston, has a sensible and timely paper on this subject in this issue. I endorse the treatment proposed and commend the paper to the thoughtful consideration of the profession.

**Dr. Daniel's Book.**—*Recollections of a Rebel Surgeon.* A second edition of this book, revised and copiously illustrated (150 new pictures in it), has just been issued by the Clinic Publishing Company, Chicago. Price, cloth, $1.00; paper, 50 cts. See advertisement.

**Medical Treatment of Cancer.**—Physicians interested in the successful treatment of cancer, as per article in the *Southern Medical Journal* for September, can obtain full information by addressing, without stamp, DR. H. B. ESMOND, West Fairlee, Vermont.

**Dr. Blunt,** State Health Officer of Texas, resigned on the 19th (October ult.), on account of ill health, and at this writing, (Oct. 21), he is said to be seriously ill. He has returned to his home at Lockhart. Dr. Jones is in charge of the office, acting State Health Officer.

---

**By the bye,** that reminds me to say to those who read this: Doctor, you received your bill in July. If you hav'nt remitted, do so now, right now. Now is your collection time. Divide. It is my collection time too. Top crop ought to be coming in by this time; that's the *doctor's crop, you know.*

---

**Dr. G. W. Christian,** of Austin, was found dead in his office on the morning of October 6th (ult.). An empty chloroform bottle and a towel on the body led to the belief that he committed suicide. Dr. Christian formerly practiced at Burnet, then at Houston, and had removed from that city to Austin within the past two years. Aged 58.

---

**Wanted.**—The name and address of every ex-Confederate Surgeon or Assistant Surgeon in Texas and adjoining States where this is read. Send it to Dr. H. A. Mosely, Dallas, Texas, Chairman Committee of Arrangements for the Annual Reunion (at Dallas) next June, of the Association of Army and Navy Medical Officers of the Confederate States.

---

**Splendid Location** for a physician in rich and prosperous section of South Texas, can be secured by purchasing residence, office, stables, etc., and a cottage on separate lot. Practice realizes $3,500 to $6,000 yearly. Price of property, $3,200; $1,000 cash, balance on accommodating terms. Address F. W., care TEXAS MEDICAL JOURNAL, Austin, Texas.

---

**New Orleans Polyclinic.**—Physicians will find the Polyclinic an excellent means for posting themselves upon modern progress in all branches of medicine and surgery. The specialties are fully taught, particularly laboratory work. Fifteenth annual session opens November 4, 1901. For further information address Dr. Isadore Dyer, Secretary, New Orleans Polyclinic, New Orleans.

---

**For Sale.**—An opening for a sober and capable physician can be had by purchasing, for $200, a small stock of drugs, in Matagorda county, in a small town. Good paying country practice of $1200 a year or more. It is near a large rice farm of 15,000 acres. The acreage will be increased and the practice will be increased, as more people will be needed at these plantations. The resident physician wishes to go to a large town. Address Dr. M. C., care of TEXAS MEDICAL JOURNAL, Austin, Texas.

---

**The Systematic Doctor.**—An excellent paper under this title, appeared in our last issue; but as a consequence of oversight or negligence in making up the forms, the name of the author was omitted. I regret it exceedingly. The omission was not discovered until too late to remedy it. The writer is DR. R. E. CARROLL, of Calvert, and the paper was read at the Calvert meeting of The Brazos Valley Medical Association, May 14th, last.

---

**The Epileptic Asylum.**—In addition to the $200,000 appropriated last session for the Epileptic Colony at Abilene, drawings of which, with a descriptive paper by Dr. Worsham, we expect to publish next month, the Legislature at the recent session appropriated $50,000 to complete the purchase of standpipe, heating apparatus and necessary machinery for power, light and water, laundry purposes, etc. The contractors and Mr. O'Connor, designer and supervising architect, are at Abilene now, and work will begin at once.

---

**A Bargain in a Fine Tieman Case.**—I have had sent me to sell one of Tieman's cases of hand made instruments for amputations and general surgery. It cost $75, and is in perfect condition. The instruments are up to date, and all have carved, (checked) genuine ivory handles. Moreover, it is a historical case, it having been used all through the Civil War by one of the foremost Confederate surgeons. It can be bought for $30, and it will be sent by express prepaid, on receipt of that amount, or sent C. O. D. with privilege of examination.—EDITOR.

---

**Attention is Called** to the "Red-Back's" large and growing advertisement patronage. It is clean and first class, cash business; no chips and whetstones; no dead beats. The "Red-Back" is nearly seventeen years old: stability. Advertising patronage held from ten to sixteen consecutive years: reliability, *it pays*, or they would drop out. That tells the tale: efficiency. The

"Red Back" has the largest and most prompt paying list of subscribers in the South: popularity; worth, vigor, originality; and its subscription list is growing like a two year old on spring oats.

---

**The State Board of Medical Examiners** held its first meeting in Austin October 1st and 2nd. Licenses were issued to only four applicants, who presented themselves for examination. One applicant was rejected. Over five hundred certificates of diplomas which had been registered since January, 1891, were sent in and licenses will be issued to the holders as rapidly as Dr. Smith, the secretary, can get them ready and mail them. Dr. Smith requests us to ask the impatient parties to let up a little on him. He is being overwhelmed with inquiries about the licenses, and says he is getting them ready as fast as he can. I am not informed as to the number of diplomas turned down. It is gratifying to state that the Attorney-General has given an opinion to the effect that the State Board of Medical Examiners is the sole judge of the character and standing of medical colleges, and can reject the diploma of any college not in good standing, registered since 1891.

---

**Nicholas Senn Prize Medal.**—The committee on the Senn Medal beg leave to call attention to the following conditions governing the competition for this medal for 1902:

1. A gold medal of suitable design is to be conferred upon the member of the American Medical Association who shall present the best essay upon some surgical subject.

2. This medal will be known as the Nicholas Senn Prize Medal.

3. The award will be made under the following conditions: *a.* The name of the author of each competing essay shall be enclosed in a sealed envelope bearing a suitable motto or device, the essay itself bearing the same motto or device. The title of the successful essay and the motto or device is to be read at the meeting at which the award is made, and the corresponding envelope to be then and there opened and the name of the successful author announced. *b.* All successful essays become the property of the Association. *c.* The medal shall be conferred and honorable mention made of the two other essays considered worthy of this distinction, at a general meeting of this Association. *d.* The competition is to be confined to those who at the time of entering the competition, as well as at the time of conferring the medal, shall be members of the American Medical Association. *e.* The competition for the medal will be closed three months before the next annual meeting of the American Medical Association, and no essays will be received after March 1, 1902.

Communications may be addressed to any member of the committee, consisting of the following: Dr. Herbert L. Burrell, 22 Newbury Street, Boston, Mass.; Dr. Edward Martin, 415 S. 15th Street, Philadelphia, Pa.; Dr. Charles H. Mayo, Rochester, Minn.

**The Transactions of the Texas State Medical Association** has just been issued from the press of Von Boeckmann, Schutze & Co., who are the State printers, and also the printers of the Red Back, and it is a beauty. For excellence in quality of paper and general get-up, typography, binding, etc., it can not be excelled by any house in New York or Chicago, and V. B., S. & Co. are not "*union*" either. There are 460 pages. It is bound in handsome brown cloth and gilt, uniform with preceding volumes under Secretary West's administration. The JOURNAL wants to express both to Dr. West and the printers its appreciation of this excellent work. The secretary is to be complimented, especially on the exhaustive cross index, which occupies twenty pages. Every subject is cross-indexed, so that it will be an easy matter to find what is sought, with little trouble. The type is clean, and the text is easily read. It will ever be a wonder to me how Dr. West can find time to do so much and such excellent work for the association and attend also to his practice and other interests. The 1901 volume of Tranactions excels any of its predecessors and will be highly prized by those who receive it. Of the contents I will speak some other time. Of the "minutes" I had a pretty full report last May. There are very few illustrated papers in this volume, and our handsome president was too modest to send the publishers his photo for an engraving; the first break in the series for many years.

